# Hierarchically structured superhydrophobic flowers with low hysteresis of the wild pansy (*Viola tricolor*) – new design principles for biomimetic materials

**DOI:** 10.3762/bjnano.2.27

**Published:** 2011-05-04

**Authors:** Anna J Schulte, Damian M Droste, Kerstin Koch, Wilhelm Barthlott

**Affiliations:** 1 Nees Institute for Biodiversity of Plants, University of Bonn, Meckenheimer Allee 170, Bonn, Germany; 2 Rhine-Waal University of Applied Sciences, Landwehr 4, Kleve, Germany

**Keywords:** anti-adhesive, petal effect, petal structures, polymer replication, superhydrophobic

## Abstract

Hierarchically structured flower leaves (petals) of many plants are superhydrophobic, but water droplets do not roll-off when the surfaces are tilted. On such surfaces water droplets are in the “Cassie impregnating wetting state”, which is also known as the “petal effect”. By analyzing the petal surfaces of different species, we discovered interesting new wetting characteristics of the surface of the flower of the wild pansy (*Viola tricolor*). This surface is superhydrophobic with a static contact angle of 169° and very low hysteresis, i.e., the petal effect does not exist and water droplets roll-off as from a lotus (*Nelumbo nucifera*) leaf. However, the surface of the wild pansy petal does not possess the wax crystals of the lotus leaf. Its petals exhibit high cone-shaped cells (average size 40 µm) with a high aspect ratio (2.1) and a very fine cuticular folding (width 260 nm) on top. The applied water droplets are in the Cassie–Baxter wetting state and roll-off at inclination angles below 5°. Fabricated hydrophobic polymer replicas of the wild pansy were prepared in an easy two-step moulding process and possess the same wetting characteristics as the original flowers. In this work we present a technical surface with a new superhydrophobic, low adhesive surface design, which combines the hierarchical structuring of petals with a wetting behavior similar to that of the lotus leaf.

## Introduction

Plant surfaces provide a large diversity of hierarchically designed structures with various functions [1–2]. Different types of epidermal cells (micro-roughness) exist in combination with cuticular folds or epicuticular waxes (nano-roughness), or both, on top [1,3]. Hierarchy in surface sculpture can cause water repellent and self-cleaning properties (“Lotus effect”) [4–6] or cause air retention under water (“Salvinia effect”) [7–8]. Superhydrophobic, self-cleaning surfaces possess a static contact angle (CA) equal to or above 150°, and a low hysteresis angle, where water droplets roll-off at surface inclinations equal to or below 10° [6,9]. One of the most important biological water repellent and self-cleaning surfaces is the lotus (*Nelumbo nucifera)* leaf [4–5]. Its water repellence is based on two factors: Surface roughness and a hydrophobic surface chemistry. The micro-morphological characteristics of lotus leaves are papillose cells covered with a dense layer of small hydrophobic wax tubules. In plants, surface waxes occur as thin films (two-dimensional waxes) or as wax tubules, platelets, rodlets or other three-dimensional waxes [1,10]. In lotus leaves, air remains trapped below a water droplet and the contact area between the water and the leaf surface is thereby minimized [1]. This micro- and nanostructured surface, composed of low surface energy materials, leads to a high CA (163°) and a low hysteresis and tilt angle (2–3°). Additionally, lotus leaves show low adhesive properties to adhering particles. Thus, contamination by dust, pollen or even hydrophilic particles such as grime are carried away by water droplets which results in a clean surface [4].

Two distinct models are proposed to explain the wetting behavior of rough surfaces. In the Wenzel model [11] roughness increases a solid surface area; this geometrically enhances its hydrophobicity. In the Cassie–Baxter model [12] air remains trapped below the droplet in the surface cavities, which also leads to a superhydrophobic behavior, because the droplet sits partially on air [13].

The Wenzel model describes homogeneous wetting by the following equation,

[1]
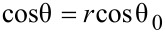


where θ is the static CA for a rough surface and θ_0_ is the static CA for a smooth surface. The surface roughness *r* is defined as the ratio of the actual over the apparent surface area of the substrate. The Cassie–Baxter model describes heterogeneous wetting by the equation,

[2]
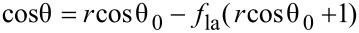


where *f*_la_ is the fraction of solid in contact with the liquid and is dimensionless.

Further important factors in surface wetting are the static contact angle hysteresis (CAH) and the tilt angle (TA). The CAH describes the difference between the advancing and receding CAs of a moving droplet, or of one increasing and decreasing in volume. The CAH occurs due to surface roughness and heterogeneity [14–15]. Low CAH results in a low TA, which describes the TA of a surface at which an applied water droplet starts to move [15].

Nowadays, transitional states between the Wenzel and Cassie–Baxter states have been discovered. Wang and Jiang [16] proposed five different states for superhydrophobic surfaces, where the lotus and gecko states are treated as special cases in the Cassie–Baxter model. Feng et al. [17] proposed a sixth superhydrophobic state, called the “Cassie impregnating wetting state” or “petal effect”. Both describe superhydrophobic surfaces with high adhesive forces to water, and this means that the wetted surface area is smaller than in the Wenzel model but larger than in the Cassie–Baxter model. Feng et al. [17] demonstrated this effect on rose flowers (petals). The surfaces of petals are often morphologically characterized by micro papillae with cuticular folds on top. In contrast to the lotus surface with air pocket formation between cell papilla, wax crystals and salient water droplets [18], the petal surface seems to prevent air pocket formation and droplets penetrate into the cuticular folds by capillary forces. It is proposed that the sizes of both micro- and nanostructures are larger than those found on the lotus leaves. Water droplets are expected to penetrate into the larger grooves of the petals, but not into the smaller ones and, thus, cause the Cassie impregnating wetting state [17].

The structure-based wetting characteristics of petals seem to offer a great alternative for the development of biomimetic superhydrophobic materials for micro droplet transport in micro fluidic systems, sensors or optical devices [19–20]. These hierarchically designed petal surfaces, with micropapillae and cuticular folds on the papillae top, can be precisely reproduced and are suitable for the industrial production in large area foil imprinting processes. In contrast, the hierarchically organized structures of the lotus leaf are composed of micropapillae with randomly distributed tubules on top. The development of such a surface architecture requires two production steps. Firstly, the microstructures must be produced by moulding, lithography or in-print-techniques. Secondly, the nanostructure production requires expensive lithographic techniques, or self-assembling materials, such as metal oxides [9,21].

Some attempts have been made to fabricate superhydrophobic surfaces with high adhesion properties inspired by rose petals [20,22–25]. Bhushan and Her [25], for example, replicated dried and thereby collapsed, micropapillae, and examined the wetting behavior of these structurally changed petals. Bormasheko et al. [24] or Shi et al. [22] fabricated “petal effect” surfaces by impregnating a polyethylene film with *Lycopodium* particles (spores) or with techniques such as electromechanical deposition of metal aggregates, which show the same wetting behavior as rose petals, but showed a different surface design than the native petals used as biological models. Xi and Jiang [23] replicated native rose petals with polydimethylsiloxane (PDMS), and fabricated surfaces that are topographically very similar to those of the original rose petals. However, their replicas possessed high adhesive forces to small (2 µl) water droplets, which cannot provide self-cleaning properties.

One simple and precise method to transfer petal surface structures into an artificial material is a soft lithography technique called replica moulding [26]. Specifically, for the replication of biological surfaces Koch et al. [27–28] introduced a cost-efficient, two-step replication technique. This precise method prevents shrinking and damaging of the biological master during the replication process by avoiding a vacuum preparation step or critical temperatures as are used in most other techniques, and biological surface structures with an extremely high aspect ratio (ar) can be replicated [29].

**Figure 1:**
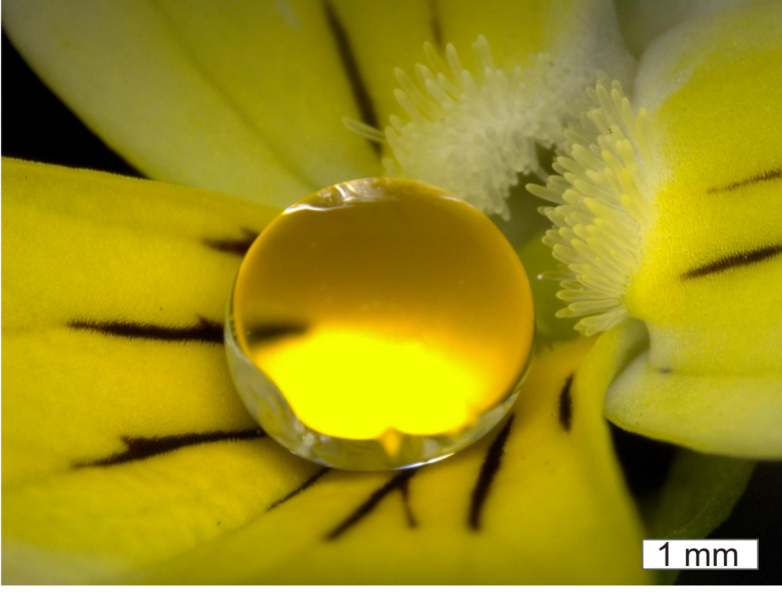
Macro photo of a water droplet on a flower of the wild pansy *(Viola tricolor)*.

In this study, we present the superhydrophobic surface of the wild pansy *Viola tricolor* (Figure 1), with a low TA and discuss the influence of papillae morphology and the dimensions of cuticular folding on the petal wetting state. To this end biomimetic replicas of four petals, differing in their surface morphology, were generated and their wetting behavior was examined by measuring the static CA and the TA. Finally, the contact area between a water droplet and the *Viola* petal surface was examined and superhydrophobic artificial petal replicas with low adhesive properties were generated.

## Results and Discussion

### Micromorphological characteristics of the surfaces

**Figure 2:**
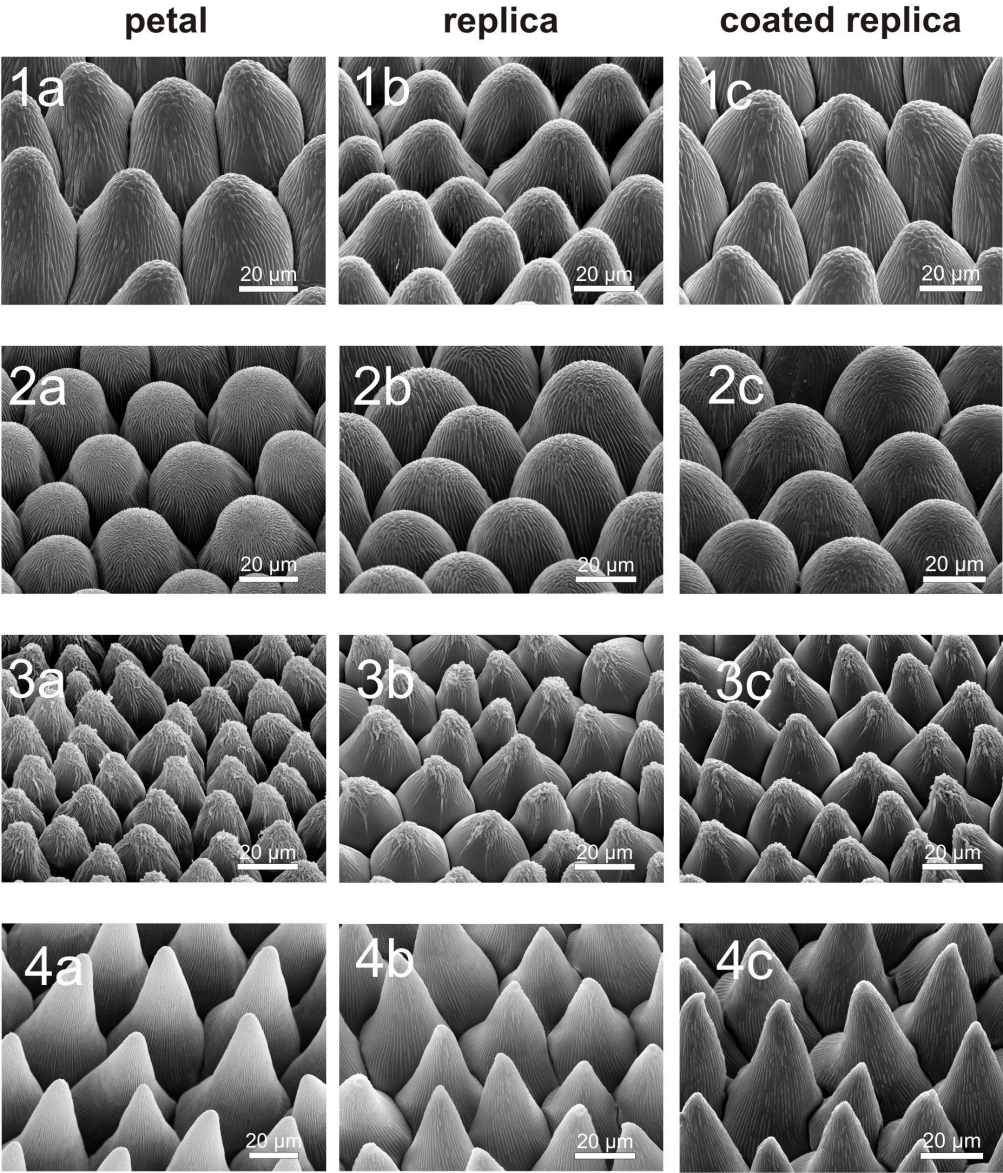
SEM micrographs of the petal surfaces (1a–4a), the uncoated polymer replicas (1b–4b) and the coated replicas (1c–4c) of *Cosmos atrosanguineus* (1a–1c), *Dahlia pinnata* (2a–2c), *Rosa chinensis* (3a–3c) and the wild pansy *Viola tricolor* (4a–4c).

Scanning electron microscope (SEM) investigations were made to characterize the micro- and nanostructures of the petals and their replicas. Petals of four different species which differ in their cell shape and dimension as well as in their wetting behavior were chosen. Figure 2 illustrates the SEM micrographs of the petal surfaces and their uncoated and coated polymer replicas [in the following the uncoated replicas are marked with a subscript *r* (*= replicas*), the coated replicas with a *cr* (*= coated replicas*) and the original petals are unmarked].

**Figure 3:**
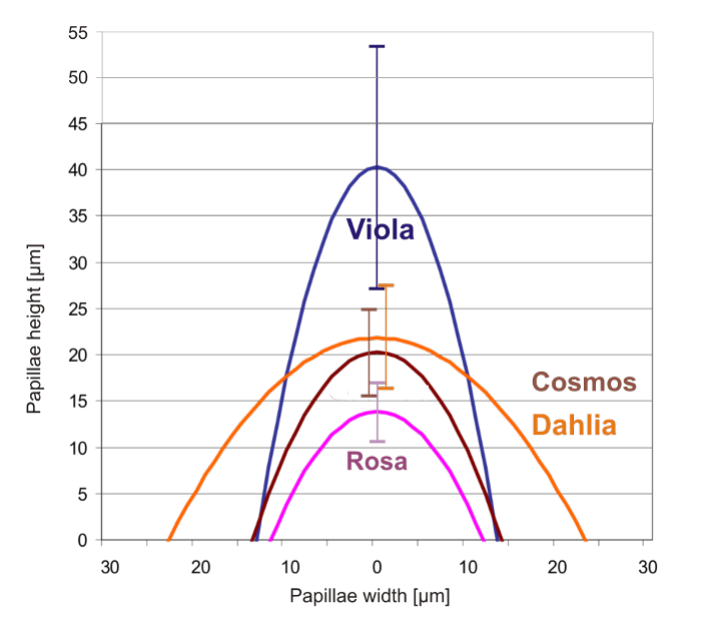
Diagram of the micropapillae dimensions of the average papilla shape on the upper surface of the *Cosmos_r_*, *Dahlia_r_*, *Rosa_r_* and *Viola_r_* petals. Also shown is the standard deviation of the papillae height (colored bars).

**Figure 4:**
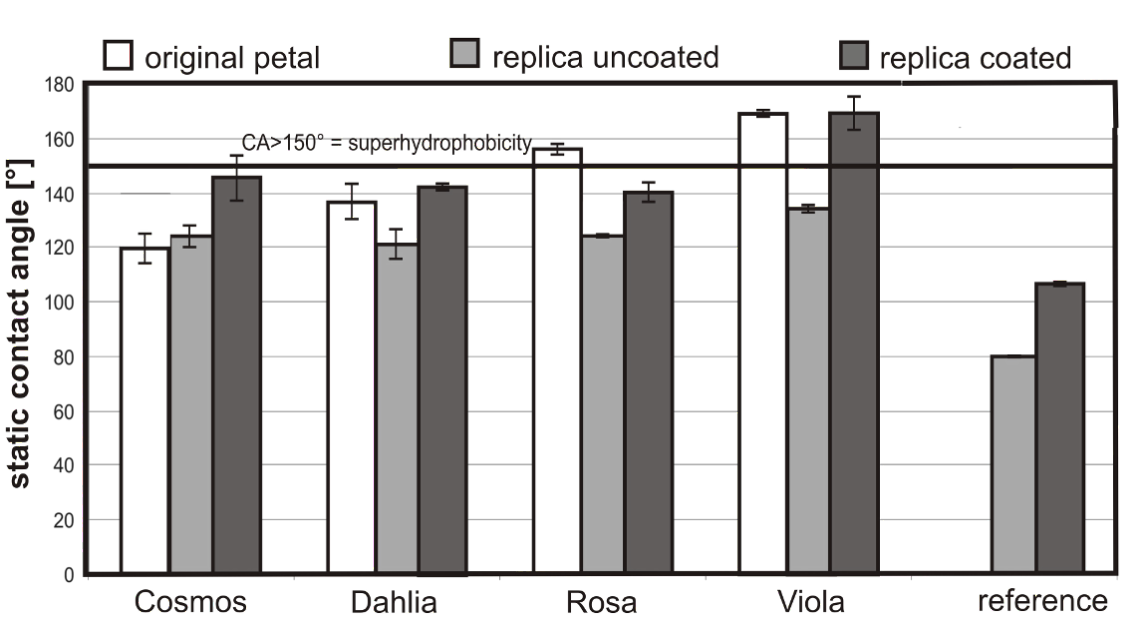
Static CAs of 5 µl water droplets on the surfaces of fresh (original) petals, their uncoated and coated polymer replicas and of the reference (uncoated and coated flat polymer; *n* = 10).

Petal surfaces of all four species are characterized by micropapillae with a cuticular folding on top (Figure 2; 1a–4a). As the pictures show, the replicas possess the same surface structures as the original petals. Minor deviations between the papillae shape of the original petals and the replicas may arise from critical point preparation of the petals (Figure 2; 1a–4a). The replicas were made from fresh turgescent flowers and the replication material used can mould a master structure to a high precision (replica deviations <2 nm from a master structure; Supporting Information File 1, Figure S1). Because of this, one may assume that the replicas display the real shape of the fresh petal surface structures. SEM pictures also show that antispread coated replicas (Figure 2; 1c–4c) possess the same surface structures as the uncoated replicas (Figure 2; 1b–4b). Accordingly, the structural parameters were collected on the uncoated replicas. Differences between the petal structures could be found in the dimensions of papillae and folds. *Rosa_r_* and *Viola_r_* are characterized by relatively sharp micropapillae (Figure 2; 3b, 4b), while *Dahlia_r_* and *Cosmos_r_* possess micropapillae with rounded tops (Figure 2; 1b, 2b). Furthermore, the micropapillae of the four different species vary from about 14 µm (*Rosa_r_*) to 40 µm (*Viola_r_*) in height, from 17 µm (*Rosa_r_*) to 33 µm (*Dahlia_r_*) in their midwidth (papillae diameter at half of the papillae height) and from 25 µm (*Viola_r_*) to 48 µm (*Dahlia_r_*) in their peak-to-peak distance (Table 1).

**Table 1:** Micropapillae characteristics of the petal polymer replicas: average values (av) and their standard deviation (σ) values are shown (*n* = 30).

Micropapillae							
Replica	Height [µm]		Midwidth [µm]		Aspect ratio (ar)	Papillae peak to peak distance [µm]	
	av	σ	av	σ	av	av	σ

*Cosmos_r_*	20.3	4.7	19.6	3.7	**1.0**	41.0	11.8
*Dahlia_r_*	21.8	5.6	32.7	4.2	**0.7**	48.4	10.1
*Rosa_r_*	13.8	3.2	16.5	3.0	**0.8**	31.1	8.9
*Viola_r_*	40.2	13.1	18.9	3.9	**2.1**	24.9	3.8

The average aspect ratio (ar) of the papillae shows similar values for the *Cosmos_r_*, *Dahlia_r_* and *Rosa_r_* papillae (ar 1.0; 0.7; 0.8). In contrast, the average ar of the *Viola_r_* papillae is much larger (ar 2.1). In this context it is noted that the standard deviation (σ) of *Viola_r_* papillae height is also higher than the standard deviation of the other species. The micropapillae dimensions are shown schematically in Figure 3.

Differences between the four species were also found in the distribution and dimensions (width and distance) of the cuticular folds (Table 2). While the micropapillae of *Dahlia_r_* and *Viola_r_* are completely covered with folds, the *Cosmos_r_* and *Rosa_r_* papillae only exhibit dense folding on top of the papillae and some single folds at the papillae side (Supporting Information File 1, Figure S2). Combinations of relatively thick folds separated by a small distance and thin folds separated by a large distance were found. The width of the folds varied from 260 nm (*Viola_r_*) to 600 nm (*Cosmos_r_*) and the distance between the single folds varied from 210 nm (*Dahlia_r_*) to 460 nm (*Cosmos_r_*).

**Table 2:** Characteristics of cuticular folds found in the replicas of the petals: average values (av) and standard deviation (σ) values of the fold width and distance in µm (*n* = 30).

Cuticular folds				
Replica	Width [µm]		Distance [µm]	
	av	σ	av	σ

*Cosmos_r_*	0.60	0.09	0.46	0.10
*Dahlia_r_*	0.39	0.08	0.21	0.06
*Rosa_r_*	0.41	0.09	0.21	0.09
*Viola_r_*	0.26	0.07	0.45	0.12

### Wettability of the petals and their replicas

Static CA and the TA measurements were performed to compare the surface structures with the wettability. Two superhydrophobic petals (*Rosa* CA 155.6° and *Viola* CA 169°) and two hydrophobic petals (*Cosmos* CA 118.3° and *Dahlia* CA 136.4°) were found (Figure 4).

The static CA of the rose petals correlates well with the CA of roses previously measured by Feng et al. [17] (CA 152.4°), Xi at al. [23] (CA 154.3°) and Bhushan et al. [25] (CA 155°). The CA of the *Cosmos* petal was only 118°, thus, the *Cosmos* surface was more hydrophilic than the other petal surfaces. Except for *Cosmos*, all uncoated polymer replicas feature a lower CA than their biological model and thus did not show the same wetting behavior. This suggests that the replica material is more hydrophobic than the cuticle of the *Cosmos* petal and more hydrophilic than the cuticles of the other species investigated. The flat uncoated polymer had a CA of 79.3°, which is by definition a hydrophilic surface [9]. With respect to the Wenzel equation (Equation 1) a CA decrease through structuring of the hydrophilic polymer was expected [11]. In contrast to that, an increase of surface roughness has led to an increase of the CA of the structured polymers. After covering the replicas with a hydrophobic fluorine polymer (CA of the flat fluorine polymer: 106.5°), the CA values increased conspicuously (Figure 4). These results emphasize that a hydrophobic material in combination with surface roughness is the basis for the fabrication of superhydrophobic surfaces.

**Figure 5:**
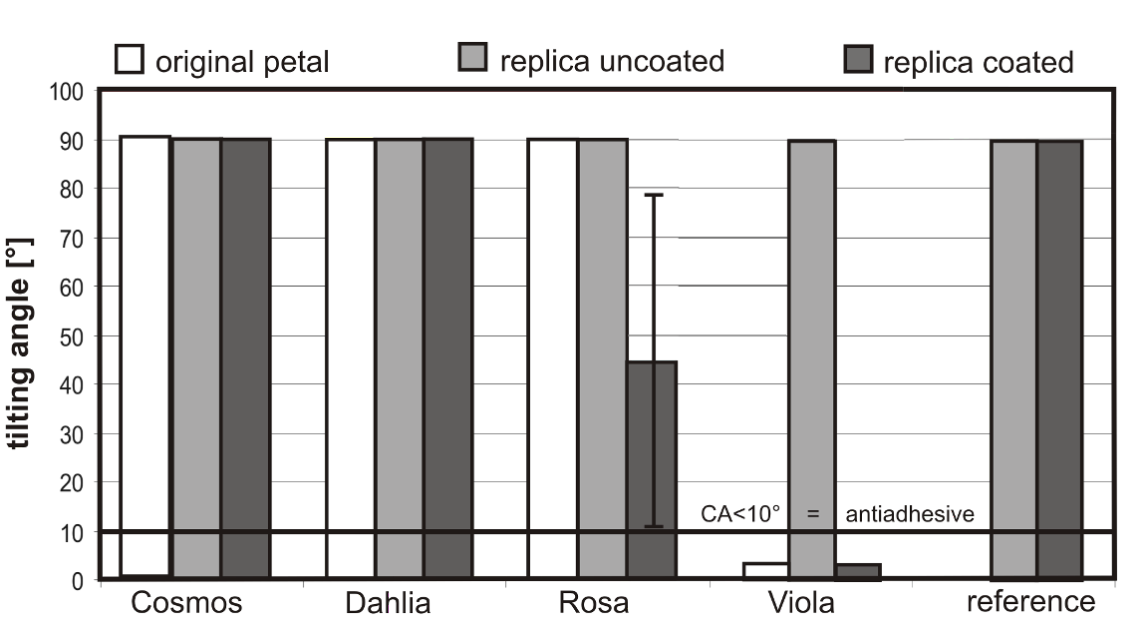
TAs of 5 µl water droplets on the surfaces of fresh (original) *Cosmos*, *Dahlia*, *Rosa* and *Viola* flowers, their uncoated and coated polymer replicas and the TA of the reference (uncoated and coated flat polymer*; n* = 10).

While the CA values of the coated replicas of *Cosmos_cr_*, *Dahlia_cr_* and *Rosa_cr_* were very similar (CA 145.2°; 141.9°; 140.0°), the CA of *Viola_cr_* was much higher (CA 168.9°). A similar tendency was found for the TAs (Figure 5). The petals of *Cosmos, Dahlia* and *Rosa* possess high adhesion to water droplets (*Cosmo*s and *Dahlia* TA >90°; *Rosa*: TA 44°), thus, water droplets do not roll-off from the petals or the coated and uncoated replicas. These data correlate well with the reported “petal effect”. Feng et al. [17] showed that *Rosa* petal surface structures impart special properties to the flowers, in that small water droplets (1–10 µl) adhere to the petals whilst larger droplets (>10 µl) roll-off. On *Viola* petals and their coated replicas, applied droplets rolled off at TAs of <5°, even when droplets with a volume smaller than 10 µl (here 5 µl) were used (Figure 5).

The TA of the coated *Rosa_cr_* was very inhomogeneous. The average TA was 44° with high standard deviation (±34.4°). *Rosa* petals possess sharp micropapillae, the folds are relatively thick (410 ± 9 nm) and the micropapillae are only 13.8 ± 3.2 µm in height. With respect to wetting stages, air pocket formation on surfaces is important. By comparison of the microstructure of *Rosa* and *Viola*, we expect larger air pocket formation in *Viola,* based on the much higher micropapilla in *Viola* (40.2 µm in height). However, in roses sometimes air pocket formation might exist because some droplets rolled-off the surface at low inclination angles (TA 10°). These observations are in contrast to those of Feng et al. [17]. Over millions of years of co-evolution, different morphological adaptations have evolved in petals. Scanning electron microscopy studies also revealed large structural variations in petal microstructures. These surface microstructures cause optical signals [30–31] or function as a tactile cue for bees [32]. For us the “petal effect” or the repellence of petals seems to be a side effect and not the primary aim of the flower. A petal is a relatively short lived organ of plants, developed for pollinator attraction, but the short duration of petal lifetime makes a self-cleaning property for pathogen defence expendable. The last point might explain why water repellence is not widespread in petals.

### Viola petals as a model for superhydrophobic, water repellent surfaces

*Viola* petals do not possess the “petal effect” and are anti-adhesive for water droplets. It is well known that hierarchical surface architecture represents optimized structures for superhydrophobic surfaces [11,33–36]. Based on the data presented here, we can describe two main superhydrophobic surface architectures for plant surfaces, the micropapillae with wax crystals [6] and micropapillae with cuticle folds. Some remarkable differences exist between the surface architecture of the lotus leaf and *Viola* petals. In *Viola* petals microstructures are larger (average height of 40.2 µm) than those of lotus leaves, which have microstructures with an average height of 15 µm [37]. The nanofolds in *Viola* have an average thickness of 0.26 µm, while the wax tubules of lotus are only 100 nm thick and ~0.5–3 µm in length [38]. Thus, the *Viola* petal possesses no three dimensional wax crystals, but a hydrophobic two dimensional wax film covering the micropapillae and nanofolds.

The distances between the structures also have an influence on the wetting stage. The average pitch value (peak to peak distances) of the lotus micropapillae is 22.6 ± 1.9 µm [37–38]. This is lower than the average pitch value of the *Rosa* micropapillae (31.1 ± 8.9 µm), but similar to the value of the *Viola* micropapillae (24.9 ± 3.8 µm). The dried rose petals investigated by Bhushan et al. [25] showed microstructures with larger pitch values than those found for the lotus leaf. On such petals water droplets seem to partially penetrate into the petal microstructures leading to a “Cassie impregnating wetting state”. The low TA found for *Viola* petals indicates a Cassie–Baxter wetting regime, in which water droplets do not penetrate into the grooves of the micropapillae. Furthermore, hysteresis can also be affected by the shape of the microstructures and adequate nano-sculpting on top. The combination of high (40.2 µm) and extremely peaked micropapillae with very fine folds (260 nm) on top apparently prevents water from penetrating into the structures by capillary force (Supporting Information File 1, Figure S2).

**Figure 6:**
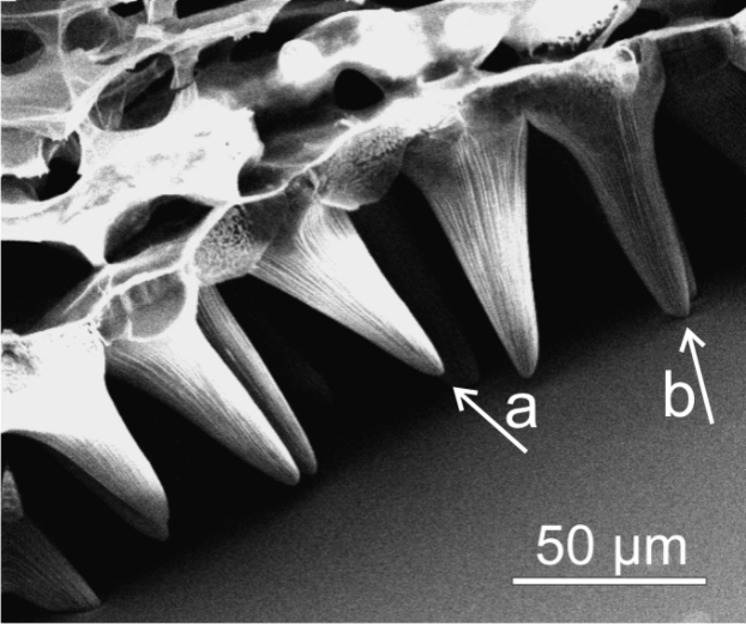
Cryo-SEM micrograph of the micropapillae of a *Viola* petal in contact with the surface of a water-glycerol droplet. Many micropapillae are not in contact with the droplet surface (a) while others are in contact with the droplet surface (b).

A high standard deviation in *Viola* micropapillae heights (σ: ±13.1 µm 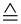 33%, Table 1) demonstrates that large variations in cell height do exist. The percentage standard deviation of the micropapillae height of the other investigated species *Cosmos_r_* (σ: ±4.7 µm 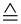 23%), *Dahlia_r_* (σ: ±5.6 µm 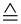 26%) and *Rosa_r_* (σ: ±3.2 µm 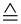 23%) is much smaller. The higher standard deviation of the micropapillae height is correlated to a large reduction in papilla contact with the applied water droplet. Cryo-SEM-investigations (Figure 6) indicated that smaller micropapillae are not in contact with the applied liquid. Thus, cell height variations further decrease the liquid–solid contact area and consequently decrease the adhesion of the liquid to the surface. Additionally, a sharp papillae tip benefits more from a lower contact area than a flat, rounded papilla tip. For the “petal effect” Feng et al. [17] proposed that water droplets penetrate into the grooves between the micropapillae. *Viola* prevents water penetration into the micropapillae grooves by reducing the papillae peak to peak distance, which is on average 24.9 µm. Much larger peak to peak distances were found in *Rosa_r_* (31.1 µm), *Cosmos_r_* (41.0 µm) and *Dahlia_r_* (48.4 µm). The results presented here show that the combination of high micropapillae with high ar, sharp tips and small peak to peak distances is required for the design of biomimetic superhydrophobic petal surfaces with low hysteresis.

The cuticular folds also have an influence on the wetting stage. On *Viola_r_* the micropapillae are completely covered with fine nano-folds (260 ± 70 nm), arranged at a separation of 450 ± 120 nm, whilst the micropapillae of the *Rosa_r_* petals are only partially covered with broader folds (410 ± 90 nm), arranged at a separation of 210 ± 90 nm. Feng et al. [17] noted folds of 730 nm width on the micropapillae with an average diameter of 16 µm and a height of 7 µm (the differences probably result from the usage of different species). Their rose petals possess a CA of 152.4° and a TA of >90°. By replicating the flowers, they developed a polymer film with a CA of 154.6° and a high adhesion to water droplets (TA >90°). Hydrophobic replicas of the *Viola* petals have a CA of 169° and a TA of <5°. These results show that finer folds arranged at small separation seem to prevent the penetration of water into the folds by capillary forces.

## Conclusion

Flower petals provide a new design strategy for the development of superhydrophobic, biomimetic materials. In contrast to superhydrophobic petals, where water droplets adhere, and which have been described before, we found a biological model (*Viola tricolor*) with a superhydrophobic, water repellent petal surface. Indeed, these flowers provide the typical surface architecture of petals (micropapillae with a folding on top), but a similar wetting behavior as that described for lotus leaves. By an easy and fast replication technique and subsequent hydrophobic coating, biomimetic replicas were fabricated. These replicas possessed the same surface structures and wettability as the biological models. The petal surface design of *Viola*, introduced here, seems to be easier and much more favourably to produce, e.g., by imprint processes, than the hierarchically organized structures which are found on the lotus leaf. In contrast to the lotus leaf structuring with randomly distributed nanocrystals the surface structures of *Viola* could be qualified, for example, for large area foil imprinting processes. Thus, a new surface design for the development of superhydrophobic, water repellent biomimetic materials is presented.

## Experimental

### Plant material

The upper surface (adaxial) sides of the petals of four different plant species were investigated. Plants were cultivated in the Botanic Gardens of the Rheinische Friedrich–Wilhelms-University of Bonn (BGB). Their scientific names are given together with their registration numbers of the BGB. Investigated species are the Chocolate Cosmos (*Cosmos atrosanguineus;* BGB 29614-8-2008), *Dahlia pinnata* (BGB 7960-9-1990), the China Rose (*Rosa chinensis;* BGB 3089-9-1979) and the Wild Pansy *Viola tricolor* (BGB 27262-4-2004).

### Fabrication of the replicas

For the fabrication of the biomimetic polymer replicas, the replication technique introduced by Koch et al. [28] was used. Here, we briefly introduce the technique and mention the modifications made. The replication technique is a two-step moulding process, in which at first a negative is generated and then a positive. For generating the negative replicas, the master (biological sample) is moulded with polyvinylsiloxane dental wax (President light body Gel, ISO 4823, PLB; Coltene Whaldent, Hamburg, Germany). In the second step, the negative replicas were filled with a two-component epoxy resin (RECKLI Injektionsharz EP, RECKLI GmbH, Herne, Germany). The use of this material is a modification in the replication process introduced by Koch et al. [28] (replication performance of the RECKLI material; Supporting Information File 1, Figure S1). After spilling the negative replicas, the epoxy resin was dried for 48 h at 25 °C. After hardening, the positive replicas were peeled off from the negative replicas and further replicas were fabricated. In total, five petals of each species were replicated and examined afterwards.

### Hydrophobization of the replicas

The replicas were dip-coated (30 sec) in a fluorine polymer (Antispread, E2/50 FE 60, Dr. Tillwich GmBH Werner Stehr) and then dried for 20 min at room temperature. Antispread is a commercially available Fluorocarbon 60 for surface hydrophobization. It forms approximately 40 nm thin layers on the substrate (producer information) and causes no additional nano-structuring on the replica surfaces. A smooth surface, dip-coated with Antispread has a static CA of 106°.

### Surface characterization

The surface structures of the biological samples and their replicas were investigated by SEM. Images were recorded using a CAMBRIDGE Stereoscan 200 SEM (Zeiss GmbH, Oberkochen, Germany), a digital image processing system (DISS 5, Version 5.4.17.0, Point Electronic GmbH, Halle, Germany) was used to visualize and measure the surface structures of the petals. Fresh plant material was dehydrated with ethanol and dried in a critical point dryer (CPD 020, Balzers Union, Balzers–Pfeifer GmbH, Aßlar). On account of their stability, the replicas did not require special preparation. All samples were sputter-coated with a 30 nm gold layer (Balzers Union SCD 040, Balzers–Pfeifer GmbH, Aßlar) prior to SEM investigations.

### Cryo-SEM examinations

To display an applied droplet in contact with the petal surface, the Cryo-SEM method, developed by Ensikat et al. [18], was used. In this method a sample–droplet (glycerol–water mixture of 1:3) complex was frozen with liquid nitrogen. A water–glycerol mixture was used as the liquid to prevent crystallization patterns on the droplet surface, which occur on pure water droplets. After this the sample was separated from the droplet (5 µl) and the surface imprint of the droplet was examined under a scanning electron microscope. All examinations were performed using a CAMBRIDGE Stereoscan 200 SEM (Zeiss GmbH, Oberkochen, Germany), equipped with a digital image acquisition system (DISS 5, Point Electronic, Halle, Germany).

### Static contact angle and tilt angle measurements

The wettability of the biological samples and the replicas was characterized by CA and TA measurements with a computer controlled goniometer OCA 30 (Dataphysics SCA 2.02, Filderstadt, Germany). Five microliters of demineralized water droplets were automatically applied to the samples via syringe and CAs were automatically determined using the Laplace–Young fitting algorithm. TAs were measured by tilting the samples (with an applied droplet on the surface) and measuring the TA at which the droplets rolled off the surface. Each measurement was repeated 10 times.

## Supporting Information

File 1 Additional figures*.*
