# Supplementary material for: Hierarchically structured superhydrophobic flowers with low hysteresis of the wild pansy (Viola tricolor) – new design principles for biomimetic materials
Source: Beilstein J Nanotechnol. 2011 May 4;2:228–36. doi: 10.3762/bjnano.2.27 (PMC3148064; doi:10.3762/bjnano.2.27)
Supplement: File 1 — Additional figures. [file Beilstein_J_Nanotechnol-02-228-s001.pdf]

Supporting Information

for

Hierarchically structured superhydrophobic flowers with low hysteresis of the wild pansy (*Viola tricolor*) – new design principles for biomimetic materials

Anna J. Schulte*[1], Damian M. Droste[1], Kerstin Koch[2] and Wilhelm Barthlott[1]

[1]Nees Institute for Biodiversity of Plants, University of Bonn, Meckenheimer Allee 170, Bonn, Germany and [2]Rhine-Waal University of Applied Sciences, Landwehr 4, Kleve, Germany

Email: Anna Julia Schulte - a.schulte@uni-bonn.de

* Corresponding author

Additional figures

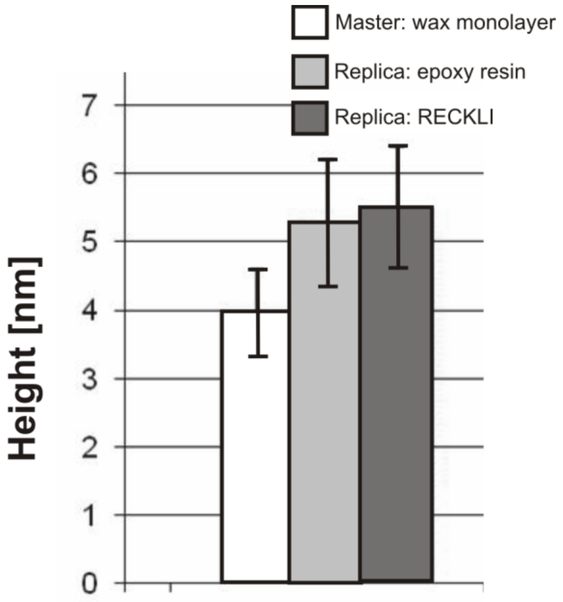

Figure S1: Replication of an octacosanol wax crystal multilayer with steps of 4 nm height. – Moulding performance of the replication material RECKLI compared to the replication performance of epoxy resin used in Koch et al. 2008 [1]. Measurements were performed by atomic force microscopy (n = 10).

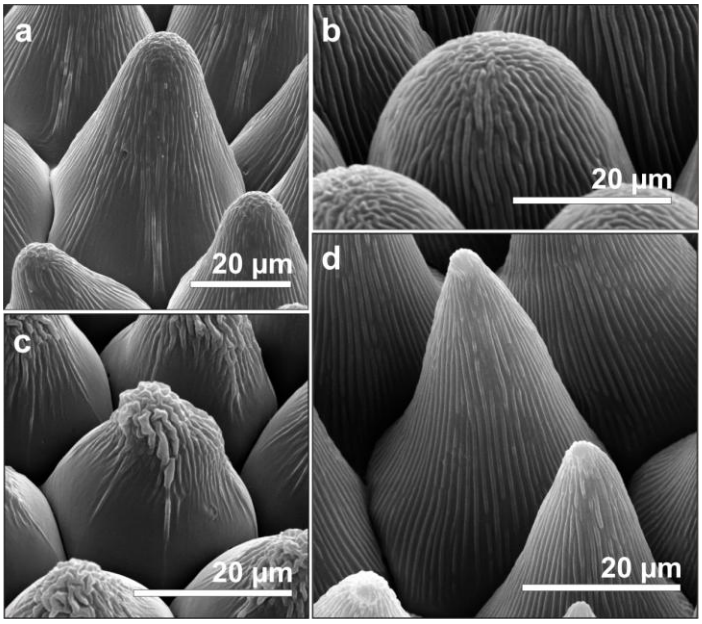

Figure S2: SEM micrographs of single epidermal cells and their cuticular folding on top: (a) *Cosmos*, (b) *Dahlia*, (c) *Rosa* and (d) *Viola*.

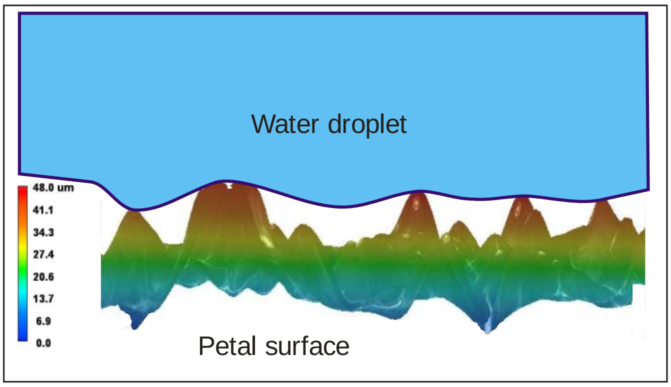

Figure S3: Schematic of the proposed wetting state of a water droplet on a 3d microscopy scan of the surface of a *Viola* petal (Cassie-Baxter state) when a water droplet is in contact with the surface of a *Viola* petal.

References

1. Koch, K.; Dommisse, A.; Barthlott, W.; Gorb, S. N. *Acta Biomater.* **2007,** *3,* 905–909. doi:10.1016/j.actbio.2007.05.013
